# Impact of GnRH agonist and GnRH antagonist on GDF9 and BMP15 expression in mouse ovaries and oocyte development

**DOI:** 10.1590/1984-3143-AR2023-0040

**Published:** 2023-12-01

**Authors:** Xin-Yu Guo, Yan Huang, Ying Ou, Xiao-Yan Chen, Ye-Xing Xian, Shi-Qin Chen, Su-Yan Xie

**Affiliations:** 1 Center of Reproductive Medicine, the General Hospital of Southern Theater Command, Guangzhou, China

**Keywords:** GnRH analogue, GnRH agonist, GnRH antagonist, GDF9, BMP15

## Abstract

GnRH analogues were widely used for controlld ovary stimulation, but their effects on oocyte quality remain contradictory. This study aimed to explore the influence of GnRH analogues on oocyte quality in mice. A total of 120 mice were randomly assigned to four groups:(i)GnRH-a+PMSG group; (ii) GnRH-ant+PMSG group; (iii) PMSG group; (iv) Control group. Ovaries were collected for quantitative real-time polymerase chain reaction (qRT-PCR) to assess GDF9 and BMP15 mRNA expression, and protein expression were evaluated by western blotting. Moreover, embryo developmental progress in vitro and implantation rate in vivo were recorded. Compared with control group, both GDF9 mRNA and protein expressions were strengthened in PMSG group, but reduced in the presence of GnRH-a or GnRH-ant. The GnRH-a group exhibited decreased BMP15 mRNA expression compared to PMSG group, while the GnRH-ant group did not show the same pattern. BMP15 protein expression were not statisticlly different among the four groups. Notably, there was no statistically difference in the expression of these two factors between GnRH-a and GnRH-ant groups. The percentage of zygotes progressing to the 2-cell stage and percentage of 2-cell advancing to the blastocyst stage were similar in the PMSG group and control group. However, both the GnRH-a and GnRH-ant groups showed decreased embryos development rates compared to other two groups. The embryonic implantation rate in control group (53.3%) was higher than that in the GnRH-a and GnRH-ant groups (33.3% and 30.8%, *P*<0.05). The difference between the PMSG (45.0%) and GnRHa group was statistically significant (*P* value of 0.023), but not between the PMSG and GnRH-ant group (*P* value of 0.486). No statistical difference was confirmed between GnRH-a and GnRH-ant groups. Our findings shed light on the safety of GnRH analogues in ovary stimulation, and highlight the need for further research to establish optimal and effective controlled ovary stimulation protocol.

## Introduction

Initialy, in vitro fertilization (IVF) procedures were performed in natural cycles without ovarian stimulation (OS). However, to obtain multiple available oocytes and increase the number of developing embryos for selection and transfer, ovulation induction became necessary. Consequently, various OS protocols have been developed, primarily relying on gonadotropin administration to induce multifollicular development. To prevent premature luteinization or ovulation, which could disrupt the collection of oocytes (Aboulghar et al., 2007; Sbracia et al., 2009), exogenous gonadotropin stimulation now involves the use of GnRH agonists (GnRH-a) or GnRH antagonists (GnRH-ant). These agents are employed to suppress the endogenous LH surge. The integration of GnRH-a or GnRH-ant in exogenous gonadotropin stimulation has facilitated the retrieval of multiple oocytes, enhancing the chances of successful IVF outcomes (Fleming, 2022).

Both GnRH-ant and GnRH-a are GnRH analogues. GnRH-a is derived from native GnRH by amino acid substitution with the extended half-life and a 100-200 times higher binding affinity for the GnRH receptors. Since its application in assisted reproductive technology in the 1980s, GnRH-a have become well accepted and is associated with an increase of pregnancy (Alama et al., 2013). GnRH-ant have been used in the field of assisted reproduction since the late 1990s. They act by directly binding the GnRH receptors and block them in a competitive manner. Thus, GnRH-ant cause an immediate, reversible, and rapid suppression of gonadotropin release (Bodri et al., 2011; Huirne et al., 2007). What has been widely accepted is that compared with GnRH-a, GnRH-ant showed shorter administration period, lower gonadotropin dosage, and lower incidence rate of ovary hyper-stimulation syndrome (Lambalk et al., 2017; Kadoura et al., 2022). But which one is the preferred GnRH analogue for IVF? Agonist or antagonist, is in dispute.

In recent years, numerous studies in humans have compared the two GnRH analogues; however, most of them have primarily focused on clinical outcomes (Lambalk et al., 2017; Rabati and Zeidi, 2012; Check et al., 2013; Trenkić et al., 2016; Yang et al., 2021; Lv et al., 2022) and endometrial receptivity (Ruan et al., 2006; Li et al., 2015). There is a lack of evidence in the literature concerning the effect of GnRH analogues on oocyte quality (Murber et al., 2009; Verpoest et al., 2017; Garcia-Ispierto et al., 2019; Uddin et al., 2023). Unfortunately, there remains a significant gap in the literature concerning the impact of GnRH analogues on oocyte quality (Murber et al., 2009; Verpoest et al., 2017; Garcia-Ispierto et al., 2019; Uddin et al., 2023), and the existing results often present conflicting findings. Thus, the objective of this study was to investigate the effects of different classical OS protocols (i.e., gonadotropins alone, GnRH-a co-treatment, or GnRH-ant co-treatment) on the biological and molecular profiles of oocytes. Additionally, we aimed to assess the developmental and implantation potential of oocytes using an IVF-mimicked mouse model.

Growth differentiation facor9 (GDF9) and bone morphogenetic protein15 (BMP15) are essential proteins secreted by oocytes in the primary follicle, playing a crucial role in organizing the surrounding granulosa and theca cells into the oocyte-cumulus-follicle complex. These two factors are also associated with cumulus cell proliferation, apoptosis, metabolism, and expansion, which are fundamental for embryo developmental competency and female fertility (D’Occhio et al., 2020; Alama et al., 2013; D' Occhio et al., 2020; Riepsamen et al., 2023). Consequently, GDF9 and BMP15 have become valuable markers used to assess oocyte quality and competency in recent research (Gong et al., 2021; Kousheh et al., 2022; Yang et al., 2020).

**Figure 1:**
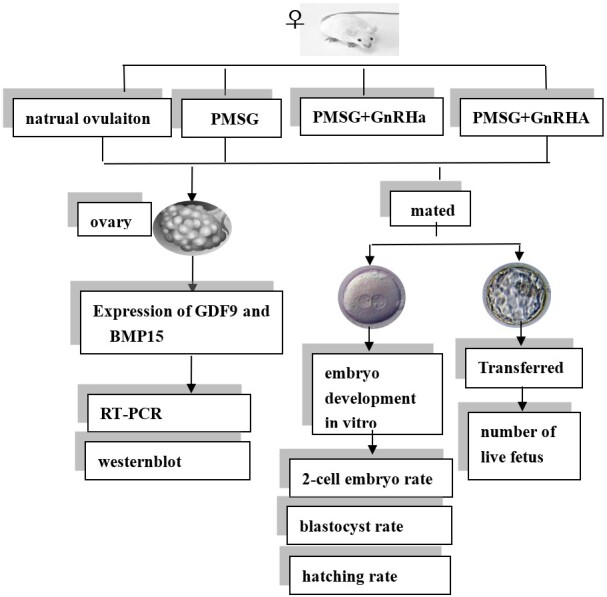
Experimental procedure.

In this study, we have identified GDF9 and BMP15 as crucial biological markers for evaluating oocyte quality. The experimental design is illustrated in Figure 1, and these findings contribute to our understanding of oocyte competence, which holds significance for enhancing fertility treatment strategies and improving female reproductive outcomes.

## Materials & Methods

### Animals

Virgin female and male SPF Kunming mice (Certificate ID:0060092/0060933, Animal Center of General Hospital of Guangzhou Military Command, Guangzhou, China) were housed under a constant 12-h light/dark cycle at 23-25°C and 50-60% humidity. The mice were fed ad libitum with a standard pellet diet and water. Estrus was identified by daily vaginal discharge and smear samples. This experiment was approved by the Committee on the Ethics of Animal Experiments of the Southern Theater General Hospital (Permit Number: SYDW2023059). A total of 120 mice exhibited regular 4-day estrus cycles were into this experiment (8- to 12-weeks old and 20-25 g body weight), which were randomly allocated to four OS groups, and the other 12 mice were used as recipients in the embryo-donation model described below.

### OS procedures

The procedures for OS performed in different groups were modified according to reference (Ruan et al., 2006): (i)GnRH-a group: GnRH-a (Decapetyl, Ferring GmbH, Germany) was i.p. injected at 1.5ug/100g bw, from day 3-11 of estrus. At 9.00 a.m. of day 9, the pregnant mare’s serum gonadotrophin (PMSG) (Intervet, USA) was i.p. injected at 40IU/100bw, followed by and injection (i.p.) of hCG (Pregnyl, Organon, Netherlands)(100IU/100g bw) 48h after the injection of PMSG; (ii)GnRH-ant group: GnRH-ant (Cetrotide, Serono, USA) was injected at 40 IU/100g bw from day 9-11 of estrum. PMSG was i.p. injected at 40IU/100g bw at 9:00 a.m. of day 9, followed by an injection (i.p.) of hCG (100IU/100g bw) 48h after the injection of PMSG; (iii)PMSG group: the mice were i.p. injected with saline at the same volume from day 3-11 of estrus and then treated with the same procedure as described for the GnRH-a and GnRH-ant groups; (iv)Control group: the mice were i.p. injected with saline only at the same volume from day 3 of estrus onwards, following the same injection schedule as described for the three groups above.

### Determination of the GDF9 and BMP15 mRNA

Five mice in each group were euthanized by dislocation shortly after hCG administration and ovaries were collected for quantitative real-time polymerase chain reaction (qRT-PCR) of GDF9 and BMP15 mRNA expression. Total RNAs were isolated by Trizol reagent according to the manufacturer’s instructions (Invitrogen, America). The quality and quantity of the RNA prepared from each sample were determined by UV absorbance spectroscopy. cDNAs were synthesized using the RevertAid™ First Strand cDNA Synthesis Kit (Fermentas, Canada). The PCR reactions were performed using Real-time PCR Detection System (ABI 7900, America). The forward and reverse primers for GDF9 were 5’-GGCCCCGCACAGGTACAACC-3’, and 5’–GCCGTACCGATGCCTGACCG–3’ (70bp). The forward and reverse primers for BMP15 were 5’-ACCGCCCTCCTTGCTGACGA-3’, and 5’-TGCGGGTCAGCCGAACGATG -3’ (143bp). The forward and reverse primers for β-actin were 5’-GAGACCTTCAACACCCCAGCC-3’, and 5’- TCGGGGCATCGGAACCGCTCA -3’ (404bp). Melting curve analysis was used to ensure the purity of the amplified PCR product. Thermal cycle conditions used as follows: 35 cycles of 95°C for 15 sec, 60°C for 60 sec using cDNA (0.25 embryo equivalent) in a final reaction volume of 25ul. The mRNA expression levels of the target genes were normalized to the expression of β-actin for mice ovaries using 2-ΔΔCt method.

### Determination of the GDF9 and BMP15 protein

Another five mice were euthanized by dislocation simultaneously and the ovaries were collected for western blotting analysis of GDF9 and BMP15 protein expression. Total proteins from the tissues were extracted by RIPA buffer containing 50 mmol/l Tris-HCl (pH7.4), 150mmol/l NaCl, 1%NP-40, 0.5%sodium deoxycholic acid, 0.1%SDS, 100ug/ml PMSF and 100ug/ml leupeptin. Samples were electrophoresed with sodium dodecyl sulphate-polyacrylamide gel electrophoresis (SDS-PAGE) using 10% polyacrylamide gels and were transferred to nitrocellulose membranes (Bio-Rad, USA). Membranes were blocked at room temperature for 1h with 5% fat-free powdered milk in TBS-T (10 mmol/L Tris, 150 mmol/L NaCl, 0.05%Tween-20, pH8.0). following three washes with TBS-T, the membranes were incubated overnight with primary antibody in 1% TBS-T at 4°C. For western blot analysis of GDF9 and BMP15, goat-anti-human polyclonal antibodies (Santa Cruz Biotechnology) at dilution of 1:250 and 1:100 were used, respectively. After incubation, the membranes were washed three times with TBS-T and then incubated with the appropriate secondary antibody at dilution of 1:2000 conjugated to peroxidase (Santa Cruz Biotechnology) at room temperature for 2 hours. Following three washes with TBS-T and three washes with distilled water, the bound antibodies were detected by enhanced chemiluminescence (ECL kit) (Santa Cruz Biotechnology). β-actin was used as an internal control to validate the amount of protein loaded onto the gels.

### Embryo collection, culture and transfer

Successfully mated female mice were identified with vaginal plugs. The day the vaginal plug presented was designated gestation day 1. Next, 18h after HCG administration, cumulus oocyte complexes were collected by flushing the removed oviducts and dispersed with 0.1% hyaluronidase (Sigma-Aldrich, St. Louis, MO). Fertilized oocytes were identified by the presence of a second polar body and two pronuclei and then cultured in human tubal fluid (HTF, Irvine Science, USA) medium supplemented with 10% human serum albumin (HSA, Vitrolife, Gothenburg, SE) at 37°C in a humidified atmosphere with 5% CO_2_. Development to 2-cell, blastocyst, and hatching blastcyst was quantified by calculating the percentage of embryos that have proceeded to the corresponding developmental stage. In the case of hatching frequency, this is specifically defined as the percentage of embryos that are partially or completely hatching at 96 hours of in vitro culture.

Ten mice in each group were put to death by cervical dislocation on gestation day 4. The horns of uterus were taken out and put into embryo culture media pre-heated under 37 and 5% CO2. Flush the uterus from broken ends near the oviduct, the blastocysts with obvious inner cell mass and blastocoele were selected under microscope and cultured in vitro.

Embryos were transferred into the uterine tubes (20 embryos per uterine tube) of separate ICR females mated during the previous night with vasectomized ICR males. Caesarian section was performed on gestation day 10 and numbers of live fetuses, moles (resorbing conceptuses) and implantation sites were scored. The implantation rate was calculated as: total number of implantation sites, moles and live fetus/number of embryos transferred.

### Statistical analysis

Data were statistically analysed by SPSS (version 25.0). The measurement data with normal distribution were expressed as mean ± standard deviation (X±s), and the differences between groups were compared by ANOVA test.The variables were expressed as median and interquartile range and the Kruskal-Wallis test was used for comparing median values of nonnormally distributed variables. The effect of different OS protocols on embryonic development and implantation rate was analysed by Chi-square test. *P-*values less than 0.05 was considered statistically significant.

## Results

### Effect of OS protocols on expression of GDF9 mRNA and protein in mice ovary

**Figure 2:**
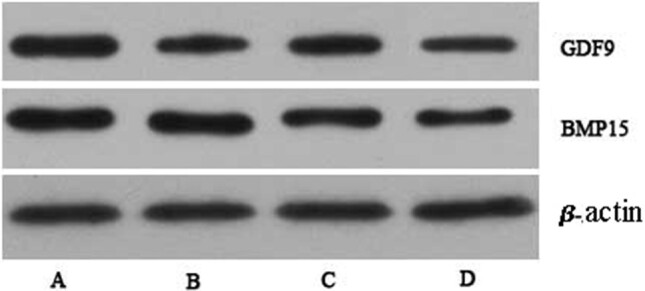
Expression of GDF-9 and BMP15 protein on mice ovaries under different stimulation protocols by western blotting. A) PMSG group; B) GnRH-a group; C) GnRH-ant group; D) control group.

**Figure 3:**
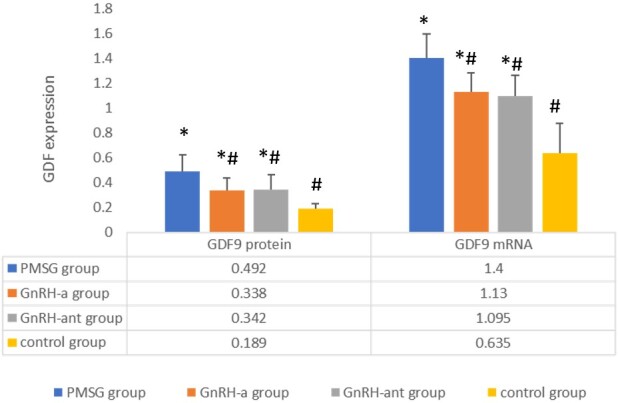
Ovarian expression of GDF9 protein and mRNA under different stimulation protocols. * Compared with control group, *P*<0.05. # Compared with PMSG group, *P*<0.05.

Both GDF9 ***mRNA*** and ***protein*** expression were strengthened in the three OS groups than those in the control group. Compared with PMSG group, GnRH-a and GnRH-ant group showed lower expression of GDF9 protein (0.338±0.098, 0.342±0.125 vs 0.492±0.135) and mRNA (1.130±0.152, 1.095±0.167, 1.400±0.194) (*P<*0.05). While no statistically significant difference of the expression of GDF9 was found between GnRH-a group and GnRH-ant group (*P*>0.05), shown in Figure 2 and Figure 3.

### Effect of OS protocol on expression of BMP15 mRNA and protein in mice ovary

**Figure 4:**
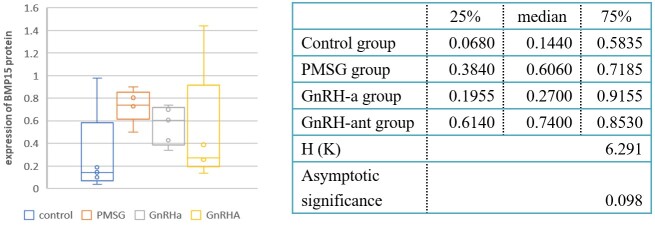
Expression of BMP15 protein by western blotting under differrent ovary stimulation protocols.

BMP15 mRNA expression detected by qRT-PCR in PMSG group (3.814±0.693) and GnRH-ant group (3.174±0.918) are higher than that in natural ovulation group (1.743±0.844). While BMP15 mRNA in GnRH-a group (2.551±0.653) was similar with that in natural ovulation group (*P*>0.05). Compared with PMSG group, GnRH-a group showed decreased BMP15 mRNA expression, however, group GnRH-ant did not have similar results. And the BMP15 mRNA expression showed no statistically significant difference between GnRH-a group and GnRH-ant group (*P*>0.05). Western-blot detection of BMP15 protein were showed in Figure 2, and statistically analysis showed no significant difference between the four group (*P*>0.05), showed in Figure 4.

### Effect of OS protocol on mice preimplantation embryos development in vitro

**Figure 5:**
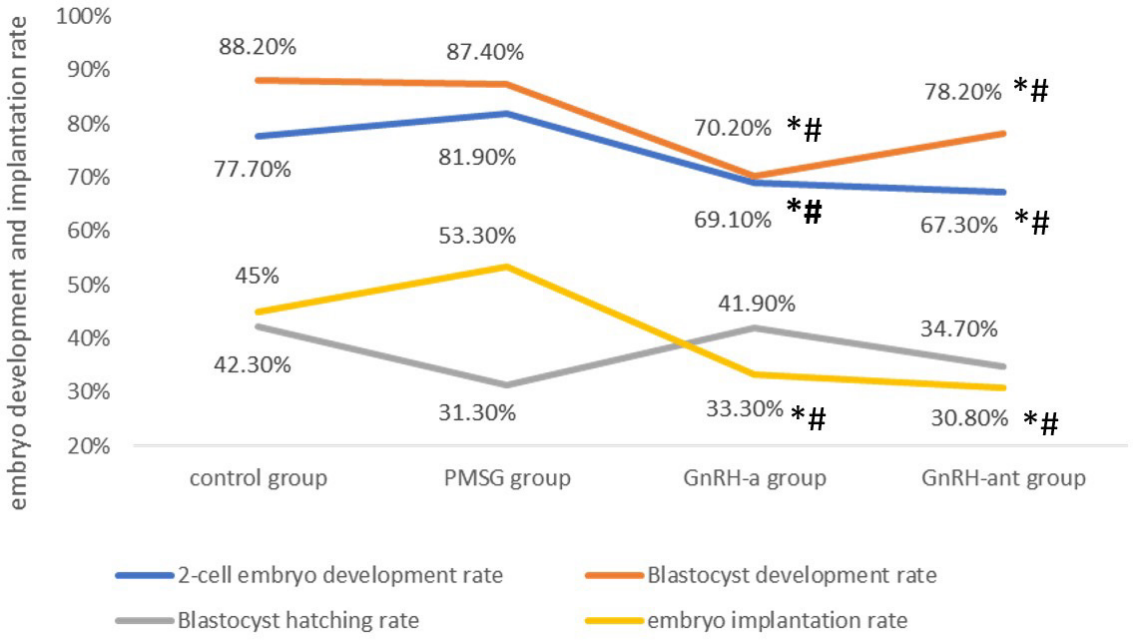
The 2-cell embyos development rate, blastocyst development rate, blastocyst hatching rate and embyos implantation under different ovary stimulation protocols. * Compared with control group, *P*<0.05. # Compared with PMSG group, *P*<0.05.

The percentage of zygotes progressing to 2-cell stage and percentage of 2-cell processing to blastocyst stage were similar in PMSG group and in control group. Both GnRH-a and GnRH-ant groups showed decreased embryos development rates compared with other two groups. Data were shown in Table 1 and Figure 5.

**Table 1:** Parameters of embryos development in under different ovary stimulation protocols.

	**2-cell embryo development rate**	**Blastocyst development rate**	**Blastocyst hatching rate**
PMSG group	81.9% (190/232)	87.4% (166/190)	31.3% (52/166)
GnRH-a group	69.1% (143/207)*#	70.2% (93/143)*#	41.9% (39/93)
GnRH-ant group	67.3% (142/211)*#	78.2% (101/142)*#	34.7% (35/101)
Control group	77.7% (127/163)	88.2% (104/127)	42.3% (44/104)

* compared with control group, *P*<0.05; #compared with PMSG group, *P*<0.05.

### Effect of OS protocol on embryonic implantation rate

In each group of the present study, 120 normal mouse embryos that reached the morula/blastocyst stage at day 4 were transferred to the corresponding mice. The number of implantation sites, moles (resorbing conceptuses) and live fetues at gestation day 10 in each group was 64 in the control group, 40 in GnRH-a group, and 37 in GnRH-ant group and 54 in PMSG group. The embryonic implantation rate in the control group (53.3%) was higher than that in PMSG group (45.0%),but the difference was not statistically significant (*P*>0.05). It was also higher than those in GnRH-a group and GnRH-ant group with statistically significance (33.3% and 30.8%, *P*<0.05). Among the three OS groups, the embryonic implantation rate in PMSG group is slightly higher than those in the other two groups, and the difference between PMSG versus GnRHa group was statistically significant (*P* value of 0.023), while the difference between PMSG versus GnRH-ant group was not (*P* value of 0.486). The embryonic implantation rate showed no statistical difference between the GnRH-a and GnRH-ant groups (*P*>0.05), seen in Figure 5.

## Discussion

GnRH analogue have been extensively utilized to prevent premature ovulation through pituitary suppression during the OS cycle. It is widely accepted that application of GnRH analogue leads to improved clinical outcomes. However, there have been only a few studies investigating the potential effects of these two GnRH analogues on oocyte quality and competency.

In the present study, we sought to investigate the potential impact of GnRH analogues on OS and oocyte quality. To contextualize our findings, it is noteworthy to mention a seminal study by Schachter in 2001 which conducted a prospective case-control evaluation for patients with a poor response to IVF stimulation (Schachter et al., 2001). Each patient actied as her own control and were compared with a standardized protocol utilizing mid-luteal administration of GnRH-a throughout the stimulation for IVF Interestingly, when GnRH-a was discontinued after 5 days of gonadotropin treatment, a benefic effect on embryo cleavage rates and morphology was observed,, suggesting a possible improvement in oocyte quality. The authors considered the efficacy of gonadotropin treatment was enhanced when GnRH-a was discontinued, hinting at a potential direct negative effect of GnRH-a on folliculogenesis and oocytes.

In the present study, GDF9 protein and mRNA expression were found strengthened in ovaries stimulated with PMSG. Proliferative granulosa cells and developed oocytes were the resource of GDF9, so the developed multiple follicles by PMSG stimulation could be the conceivable producer. These results were consistent with the report of Wei LN, and they found the controlled ovarian stimulation can promote the expression of GDF9 and BMP15 both in oocytes and GCs from normal ovulatory women (Azizollahi et al., 2021). The present study also found that, compared with the PMSG administration only, the addition of GnRH analogues, both GnRH-a and GnRH-ant reduced the expression of the GDF9, which may subsequently impair cytoplasm maturation and lead to poor oocyte developmental and implantation potency. This was supported by our *in vivo* and *in vitro* observations, GnRH-a and GnRH-ant OS showed impaired embryo developmental and implantation potency than PMSG stimulation and natural ovulation, which were not even reported.

However, unlike GDF9 expression, BMP15 mRNA expression was not affected by the administration of GnRH-ant, but decreased by GnRH-a, and the BMP15 protein expression showed no difference between the four OS protocols. In humans, there is evidence that GDF9 is expressed throughout folliculogenesis, increasing from primordial through to preovulatory oocytes, while BMP15 is primarily derived from oocytes of small antral follicles (Kristensen et al., 2014; Zhang et al., 2018). Therefore, GDF9 may be more reflective of the ovarian reserve than BMP15. A study using proteomic analysis also reported low abundance of BMP15 than GDF9 in human oocytes (Virant‐Klun et al., 2016). This may be one of the possible explanation for the differences results of the two factors in this study. In addition, the statistical analysis is calculated based on the number of samples, so the low number may have influenced the results.

There are some literature regarding the comparison of the effects of the two analogues on oocyte quality. Prospective randomized controlled trial comparing GnRH-ant versus GnRH-a in OS for PGD showed a higher clinical pregnancy rate in the GnRH-a group, but no difference in number of embryos of sufficient quality for biopsy and the number of embryos available of top quality (Verpoest et al., 2017).

681 oocytes obtained the overall incidences of oocyte dysmorphism in both the GnRH-a and GnRH-ant groups were similar, suggesting that any beneficial effects were associated with the protocols used for OS.(Cota et al., 2012). from the antagonist protocol and the agonist protocol were observed for morphology, and there was no difference between the two protocols (Cota et al., 2012). A recent study compared the effect of pituitary suppression regimens on oocyte morphology drew different conclusions. Antagonist cycles presented lower incidence of dark cytoplasm, Smooth endoplasmic reticulum (SER cluster), and ZP defects. Compared to the GnRH-a treatment, GnRH-ant's inhibitory resulted in improved oocyte maturity and morphology, despite similar laboratory and clinical outcomes (Zanetti et al., 2020).

Some other reports also suggested that GnRH-ant protocol might be a more appropriate choice than GnRH-a protocol. For the first time, the expression levels of genes involved in the cytoplasmic maturity (BMP15, GDF9), adenosine triphosphate production, and antiapoptotic process, in GV oocytes were found significantly higher in the GnRH-ant group than in the GnRH-a group (Hoseini et al., 2014). Gene expression of cumulus cells was evaluated and compared between the two GnRH analogues in another article. Bax expression in the GnRH-a group was remarkably higher than GnRH-ant group, while the mRNA expression of BCL-2 and ALCM genes were considerably greater in the antagonist protocol (Azizollahi et al., 2021).

Yet there are also favourable findings for GnRH-a,in a retrospective cohort study of 550 early miscarriage patients was conducted in 2022, higher aneuploidy rate in early aborted tissues(48.51% vs. 64.19%) and blastocysts(39.69% vs. 52.27%) were found in GnRH-ant protocol than in GnRH-a protocol. Furthermore, the blastocyst aneuploidy rate in the GnRH-ant protocol group was higher only in young and normal ovarian responders. These results should be confirmed in a multicenter, randomized controlled trial (Wang et al., 2022).

In the present study, we investigated the expression of GDF9 and BMP15 in ovaries stimulated with PMSG, which resulted in a strengthening of both factors. The multiple follicles developed during PMSG stimulation appeared to be the primary source of GDF9 and BMP15 production, originating from proliferative granulosa cells and developed oocytes. These findings align with a previous report by Wei LN, who demonstrated that controlled ovarian stimulation promotes the expression of GDF9 and BMP15 in both oocytes and GCs from normal ovulatory women (Wei et al., 2013).

However, our study revealed interesting observations when comparing PMSG administration alone with the addition of GnRH analogues, both GnRH-a and GnRH-ant. The presence of GnRH analogues led to a reduction in the mRNA and protein expression of GDF9 and BMP15. This downregulation may subsequently impair cytoplasm maturation and result in poor oocyte developmental and implantation potency.

While compared with the PMSG administration only, the addition of GnRH analogues, both GnRH-a and GnRH-ant reduced the expresssion of the mRNA and protein of these two factors, which may subsequently impair cytoplasm maturation and lead to poor oocyte developmental and implantation potency.

The present study indicated that OS with both GnRH-a and GnRH-ant might lead a negative impact on oocyte developmental potency. Though the difference between the two GnRH analogues is not distinct, GnRH-ant appeared to show a slight advantage with minor damage in the expression of BMP15 mRNA and embryo implantation rate. However, it is essential to acknowledge the limitations of this study. The experiment was performed only in mice and the dosage of GnRH analogues administration was relatively simple. With respect to the evaluation of GnRH analogues on the oocyte developmental and implantation potency, the dosage of GnRH analogue should be considered. Additionally, the sample size in our study was insufficient to allow conclusions, necessitating larger clinical studies to elucidate this controversial subject.

## Conclusion

Despite these limitations, our results still provided valuable insights into the safety of GnRH analogues in OS protocol for IVF, considering their widespread usage in these procedures. By highlighting the potential impact of GnRH analogues on oocyte quality and developmental outcomes, our study contributes to the ongoing discussions surrounding the optimization of IVF treatment protocols. Nonetheless, further research is imperative to establish a comprehensive understanding of the effects of GnRH analogues on oocyte development and implantation potency in different clinical settings.
